# Case report: Atypical spindle cell/pleomorphic lipomatous tumor masquerading as a myxoid liposarcoma or intramuscular myxoma

**DOI:** 10.3389/fonc.2022.1033114

**Published:** 2022-11-10

**Authors:** Jiro Ichikawa, Tomonori Kawasaki, Hiroki Imada, Satoshi Kanno, Naofumi Taniguchi, Tomoyuki Ashizawa, Hirotaka Haro

**Affiliations:** ^1^ Department of Orthopaedic Surgery, Interdisciplinary Graduate School of Medicine, University of Yamanashi, Chuo, Yamanashi, Japan; ^2^ Department of Pathology, Saitama Medical University International Medical Center, Hidaka, Saitama, Japan; ^3^ Department of Pathology, Saitama Medical Center, Saitama Medical University, Kawagoe, Saitama, Japan

**Keywords:** atypical spindle cell/pleomorphic lipomatous tumor, differential diagnosis, magnetic resonance imaging, histopathology, immunohistochemistry, fluorescence *in situ* hybridization

## Abstract

Atypical spindle cell/pleomorphic lipomatous tumors (ASPLTs) were recently categorized as benign lipomatous tumors. However, accurate and complete preoperative diagnosis of ASPLTs may be difficult. Furthermore, diagnosis based on magnetic resonance imaging (MRI) findings is uncertain because of the varying ratios of the fat component within the tumor. Here, we report a case of ASPLT masquerading as a myxoid tumor. Although MRI findings were consistent with a myxoid liposarcoma, needle biopsy findings suggested a myxoma, and we performed marginal resection. Histopathological findings revealed infiltrating spindle cells with atypia. In addition, immunohistochemistry (IHC) showed positive staining for CD34 and heterogeneous retinoblastoma deficiency, and fluorescence *in situ* hybridization (FISH) showed no amplification of mouse double minute 2 homolog and no rearrangement of *FUS* or *EWSR1*. When MRI and histopathological findings suggest a myxoid tumor, IHC and FISH should be considered and performed for a precise and accurate diagnosis.

## Introduction

Dei Tos et al. were the first to describe atypical spindle cell/pleomorphic lipomatous tumors (ASPLTs), which are similar to spindle cell liposarcomas, well-differentiated liposarcomas, and atypical spindle cell lipomas (1). Further molecular analysis showed that ASPLTs could be further subclassified as atypical spindle cell lipomatous tumors and atypical pleomorphic lipomatous tumors, which are benign lipomatous tumors (2). Although histopathological findings are essential for diagnosing ASPLT, diagnosis is complex because of the presence of varying proportions of atypical spindle cells, adipocytes, lipoblasts, and multinucleated cells and an extracellular matrix that consists of varying proportions of myxoid and collagenous components (3). Therefore, the differential diagnosis of ASPLT may encompass a broad range of conditions, from fatty tumors to fibrous tumors. Accordingly, histopathology, immunohistochemistry (IHC), and fluorescence *in situ* hybridization (FISH) are indispensable for an accurate diagnosis (4, 5). Although there have been few reports on the magnetic resonance imaging (MRI) findings of ASPLT, ASPLTs have varying T1 intensities, which may reflect their pathological diversity (6–8). Herein, we report a case of ASPLT masquerading as a myxoid tumor and review the pathological and MRI findings of ASPLT.

## Case description

**Figure 1:**
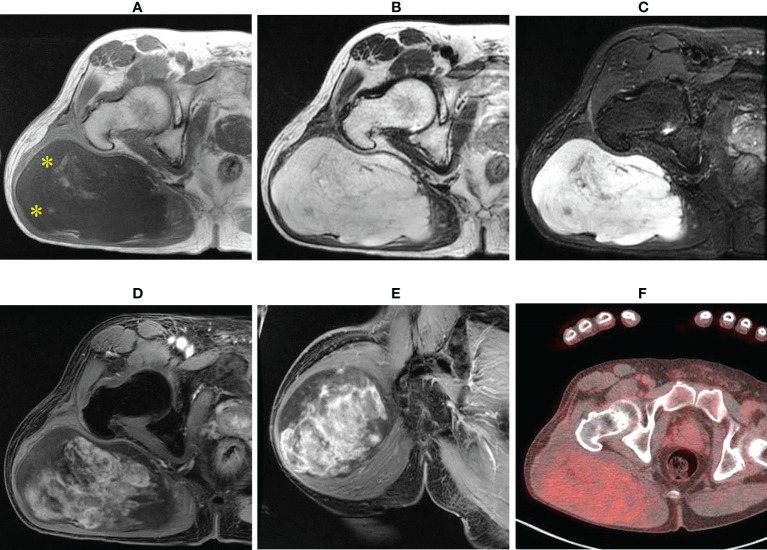
Imaging findings. T1-weighted axial images showing low and slightly high signals (yellow asterisk) **(A)**; almost homogeneous high signal on T2-weighted images **(B)** and short tau inversion recovery images **(C)**; and axial and central enhancement on a gadolinium-enhanced T1-weighted axial image **(D, E)**. Axial fluorodeoxyglucose (FDG) positron emission tomography-computed tomography (PET-CT) showing a hypometabolic tumor **(F)**.

**Figure 2:**
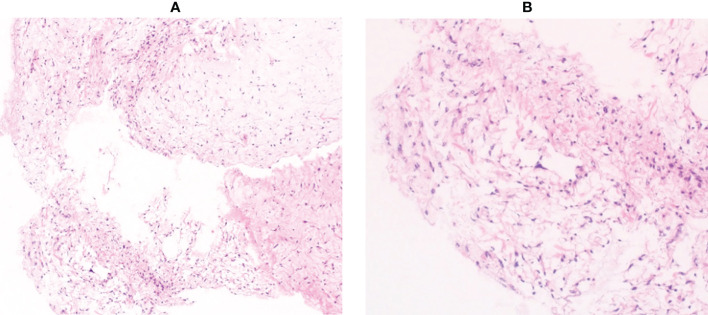
Histological findings of the needle biopsy specimen. Pathological examination showed mild proliferation of spindle cells with few atypical cells in a myxoid background (**A**, ×100 **B**, ×200).

**Figure 3:**
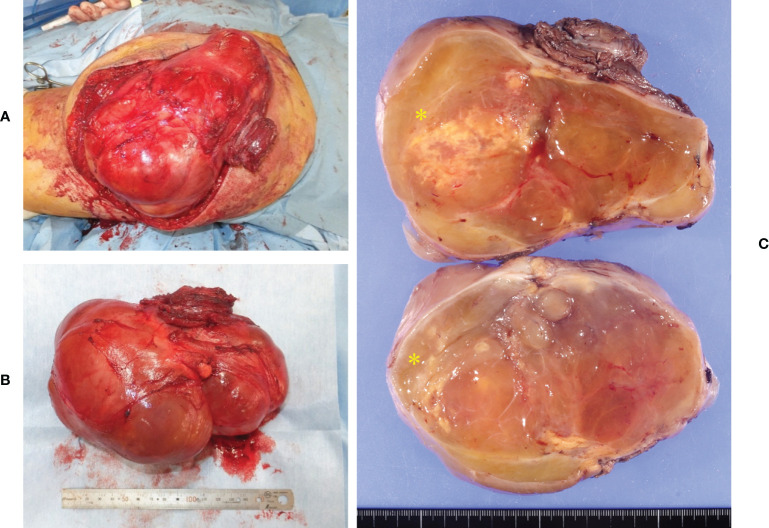
Intraoperative and pathological findings of the resection specimen. The tumor was located inside the gluteus maximus **(A)**. Macroscopically, the tumor measured 16 × 15 × 12 cm in size **(B)**. The tumor consisted of large myxoid components and small fatty components (yellow asterisk) **(C)**.

**Figure 4:**
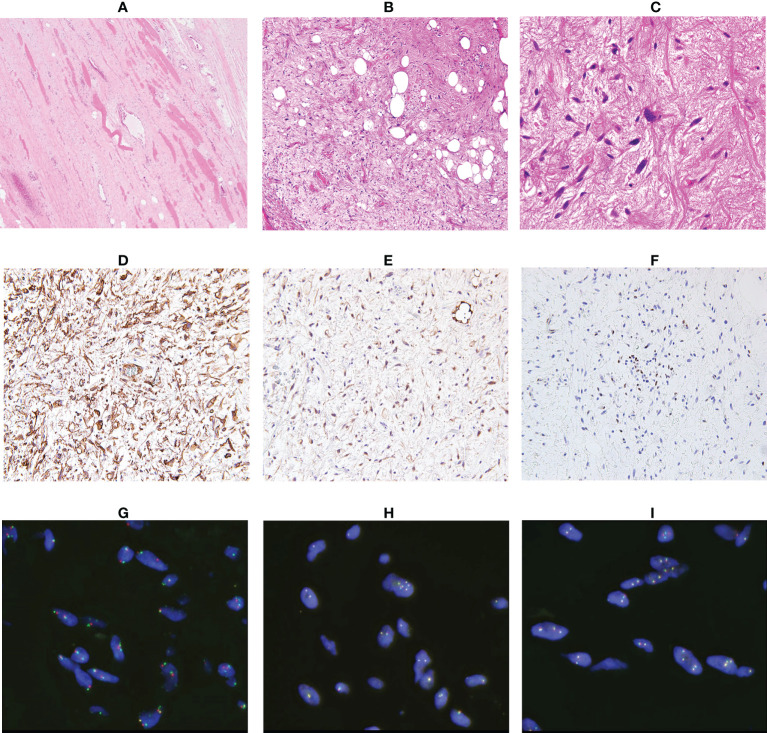
Analysis of the resected specimen. The resected specimen showed an ill-defined border (**A**, ×100) and a myxoid and fibrous lesion with spindle cells; some spindle cells had cytological atypia (**B**, ×100 **C**, ×200). Immunohistochemical results for CD34 **(D)**, S100 **(E)** and RB1 **(F)** (×200). Fluorescence *in situ* hybridization indicated no amplification of MDM2 **(G)** and no rearrangement of FUS **(H)** or EWSR1 **(I)**.

A 64-year-old man presented with a mass on his right buttock. The mass had been apparent for 1 year and was painless and gradually increased in size. Physical examination revealed no pain, swelling, or numbness. Moreover, the Tinel sign was negative, and the hip range of motion was normal. Furthermore, there was no gait disturbance. MRI revealed a low-intensity signal on T1-weighted images with a few high-intensity signals that suggested the presence of intratumoral fat (**Figure 1A**). High signal intensity on T2-weighted images (**Figure 1B**) and T2 short tau inversion recovery images (**Figure 1C**) suggested the presence of myxoid components. Heterogeneous enhancement was noted on gadolinium-enhanced T1-weighted images (**Figures 1D, E**). Positron emission tomography-computed tomography (PET-CT) showed slight ^18^F-fluorodeoxyglucose uptake in the right buttock mass, with an SUVmax of 2.8 (**Figure 1F**). The imaging findings suggested myxoid liposarcoma (MLS). However, needle biopsy findings suggested myxoma, because there were few atypical spindle cells (**Figures 2A, B**). Collectively, the preoperative findings indicated intramuscular myxoma; therefore, marginal resection was performed. The tumor was located within the gluteus maximus (**Figure 3A**). Macroscopically, the tumor measured 16 × 15 × 12 cm in size and consisted of mainly myxoid components with little fat tissue (**Figures 3B, C**). Histopathologically, the tumor infiltrated the muscle and had an ill-defined border (**Figure 4A**). While most of the tumor cells were bland and short-spindled with a myxoid matrix, some of the cells showed cytological atypia and pleomorphism. Mitosis was rare. In addition, there were areas with fatty lipid and lipoblast components (**Figures 4B, C**). IHC indicated that the tumor was positive for CD34 (**Figure 4D**) and S100 (**Figure 4E**), negative for retinoblastoma (RB1) (**Figure 4F**), and negative for desmin, estrogen receptor, Muc4, and BCL2 (data not shown). FISH showed no amplification of mouse double minute 2 homolog (*MDM2*) (**Figure 4G**) and no rearrangement of *FUS* (**Figure 4H**) or *EWSR1* (**Figure 4I**). A final diagnosis of ASPLT was made based on of the IHC and FISH results and the imaging and pathological findings.

Considering the clinical features of the tumor, we decided to conduct follow-up using imaging studies. The patient was free of recurrence 1 year after surgery.

## Discussion

ASPLT was originally reported as a spindle cell liposarcoma, a well-differentiated liposarcoma, and an atypical spindle cell lipoma (1). Although ASPLT was initially considered a subtype of atypical lipomatous tumor/well-differentiated liposarcoma (ALT/WDL), further analysis of clinicopathologic characterizations led to separate categorizations (9). Marino-Enriquez et al. suggested that ASPLTs were atypical spindle cell lipomatous tumors (4). However, analysis of the genetic and pathological features of atypical pleomorphic lipomatous tumors (10) led to classifying both atypical pleomorphic lipomatous tumors and atypical spindle cell lipomatous tumors as ASPLTs, which are categorized as benign lipomatous tumors (2). Although the exact mechanism of ASPLT tumorigenesis has not been elucidated, a subset of cases showed a loss of *RB1*, which comprises the 13q/RB1 family of tumors (2, 11).

The differential diagnosis of ASPLT based on histopathology is broad, ranging from benign to malignant lipomatous tumors as summarized in **Table 1** (3). The characteristics of both ASPLT and other tumors highly overlap, implying that differential diagnosis is very complex. Thus, IHC and FISH, in addition to histological examination, are essential for the correct diagnosis of ASPLT.

**Table 1:** Differential diagnosis of atypical spindle cell/pleomorphic lipomatous tumor.

	ASPLT	SCL	ALT/WDL	DDLS	PLS	MLS	Myxoma
**Clinical**							
Age (decade)	6th	5th to 6th	4th to 6th	5th to 6th	7th	4th to 5th	4th to 7th
Location	Superficial > deep	Superficial	deep	deep	deep	deep	deep
Size (mean or median)	5cm	<5cm	> 10cm	>10cm	8-10cm	8- 12cm	< 10 cm
**Histology**							
Atypical spindle cells	yes	no	possible	possible	possible	no	no
lipoblasts	common	rare	possible	possible	yes	rare	no
**Immunohisto-Chemistry**	Rb loss (50-70%)CD34 (60-70%)	Rb loss CD34	MDM2/CDK4	MDM2/CDK4	NA	NA	CD34S100(-)
**Molecular features**	*Rbl* deletion	*Rbl* deletion	*MDM2* amplification	*MDM2* amplification	NA	*FUS/EWSR1::DDIT3*	*GNAS* mutation

ASPLT, atypical spindle cell/pleomorphic lipomatous tumor; SCL, spindle cell lipoma; ALT/WDL, atypical lipomatous tumor/well differentiated liposarcoma; DDLS, dedifferentiated liposarcoma; PLS, pleomorphic liposarcoma; MLS, myxoid liposarcoma; RB, retinoblastoma; MDM2, mouse double minute 2 homolog; CDK4, cyclin dependent kinase 4. NA; not applicable.

The distinctive differences in clinical features between ASPLT and spindle cell lipoma (SCL) are sex and anatomic location (4, 12). Approximately 60% of patients with ASPLT are men, and most are in their 50s. With regard to tumor location, 60% of tumors occur in the limbs and limb girdles and approximately 60% occur in subcutaneous tissues. The average tumor size is approximately 5 cm (4). However, the size, depth, and anatomical location of the tumor in our case were different from those of typical ASPLT cases. There are several histological differences between SCL and ASPLT, including the presence of atypical spindle cells, atypical multivacuolated lipoblasts, bizarre pleomorphic cells, and mitotic activity. Floret-like multinucleated cells and ropey collagen are typical of SCL. These findings are uncommon in ASPLT, but they have been observed previously (3, 11). SCL and ASPLT share similar immunohistochemical features, such as CD34 positivity and the loss of RB1 (11). Although these features are observed in almost all SCL cases, ASPLT has about 50–70% positive expression of CD34 and about 60–70% RB1 loss (4, 13). In addition, although 40% of ASPLT cases are positive for S100, 90% of SCL cases have no S100 expression (4, 14). Nonetheless, ASPLT and SCL have many overlapping histological and IHC findings. Therefore, extreme caution is required during diagnosis.

Differentiating ALT/WDL, dedifferentiated liposarcoma (DDLS), and ASPLT is clinically important. The expression of MDM2 and CDK4 in IHC are key findings (3). ALT/WDL and DDLS have both MDM2 and CDK4 expression, whereas ASPLT is usually negative for both markers (3). However, care should be taken because some cases of ASPLT have shown CDK4 or MDM2 positivity (13). In these cases, no positivity may be observed in both MDM2 and CDK4, and no amplification of *MDM2* is observed using FISH (4, 13).

The presence of pleomorphism, high mitotic activity, and tumor necrosis and the lack of floret-like multinucleated cells are important for differentiating pleomorphic liposarcoma (PLS) from ASPLT (10). PLS has no characteristic IHC or FISH findings. Various MRI findings of PLS have been observed because of necrosis and intratumoral bleeding, making diagnosis based on MRI difficult (15, 16). In our case, myxoid liposarcoma (MLS) and myxoma were strongly suspected based on MRI findings. To differentiate ASPLT from MLS, confirmation of *FUS* and *EWSR1* rearrangements using FISH is required (17). Additionally, histological and morphological findings may be useful for differentiating these tumor types, as myxoma is positive for CD34 and negative for S100 and desmin (18). Therefore, complete pathological assessment, IHC, and FISH should be performed to obtain a correct diagnosis.

Previous reports of ASPLT have mainly analyzed pathological findings with no analyses of imaging findings, especially those of MRI (4, 5, 10, 11, 13). In general, the T1 intensity on MRI is important for differentiating lipomatous tumors (16); therefore, we focused on the intensity of T1-weighted images (only CT was available in one case). Based on the results of three previous cases, the T1 intensity of ASPLT can be divided into three groups: (i) high (7), (ii) high and low (6), and (iii) low (8). ASPLT and SCL tend to have similar enhancement patterns on T1-weighted images, which reflect their similar pathological findings (19). In our case, MRI showed a low intensity on T1-weighted images (pattern iii) and a high intensity on STIR images, which is caused by fat tissue within the muscle. These imaging findings suggested MLS and intramuscular myxoma rather than ASPLT. The MRI findings of myxoma and MLS include hazy, nodular internal enhancement, the presence of fat, and the tumor size (20). Based on these features, the MRI findings in our case were consistent with MLS. For tumors with high T1 intensity, ALT/WDL should be considered, and for tumors with high and low T1 intensity, DDLS should be considered. When differentiating ASPLT from ALT/WDL and DDLS, diagnosis using only MRI is nearly impossible; therefore, histology, IHC, and FISH are required for a definite diagnosis.

This is the first case of ASPLT for which positron emission tomography-computed tomography was reported. However, we did not find PET-CT useful in differential diagnosis because the SUVmax and SUC were similar to those of myxoma and MLS (20).

Generally, precise preoperative diagnosis as either benign or malignant soft tissue tumor is important because wide resection is essential for malignant soft tissue tumors and, in addition, radiotherapy and chemotherapy are needed for some histological types. These are the reasons why we should differentiate MLS from myxoma and ASPLT. In our case, preoperative imaging findings suggested MLS rather than myxoma. Notably, wide resection is needed for MLS and ASPLT, while marginal resection is sufficient for myxoma. In addition, MLS has radiosensitivity, suggesting that preoperative radiotherapy for MLS is advantageous in reducing both tumor size and local recurrence. The preoperative diagnosis using needle biopsy was intramuscular myxoma; however, the presence of spindle cells with atypia could not be confirmed. We should consider ASPLT when myxoid tumors, including myxoid liposarcoma and myxoma, are suspected based on imaging findings and needle biopsy. Although the recurrence rate is 10–15% in ASPLT cases with incomplete resection, no cases of metastasis or dedifferentiation have been reported (2, 4). In contrast, MLS has a high recurrence rate of 60% with marginal resection and metastasis rate of 30–60% (17, 21). Considering the differences in clinical features between ASPLT and MLS and although additional surgery or radiotherapy was performed in case of MLS, we decided to conduct a follow-up of our ASPLT case using imaging studies. Follow-up is essential in cases like ours, and long-term follow-up is preferable because the interval of recurrence has been reported to be 6 months to 17 years (4).

## Conclusion

Here, we present an atypical case of ASPLT and the difficulties in its differential diagnosis. Although the combination of imaging and pathological findings is essential for the precise diagnosis of soft tissue tumors, pathological findings are more advantageous than imaging findings for ASPLT because MRI findings are heterogeneous. If benign or malignant lipomatous tumors cannot be accurately diagnosed by histology, IHC and FISH should be performed. A wide resection is recommended; however, there will be some cases, such as our case, where preoperative diagnosis is inconclusive. Because ASPLT is a benign tumor that does not metastasize, careful, long-term follow-up may be more beneficial than additional surgery.

## Data availability statement

The datasets presented in this study can be found in online repositories. The names of the repository/repositories and accession number(s) can be found in the article/supplementary material.

## Ethics statement

Written informed consent was obtained from the individual(s) for the publication of any potentially identifiable images or data included in this article.

## Author contributions

Conception/design: JI, TK, and HI. Provision of study material or patients: JI, NT, TA, and HH. Data collection and analysis: JI, TK, HI, and SK. Manuscript writing: JI, TK, and HI. All authors contributed to the article and approved the submitted version.

## Acknowledgments

The authors thank Ms. Kahori Sano and Azusa Sakamoto for secretarial assistance.

## Conflict of interest

The authors declare that the research was conducted in the absence of any commercial or financial relationships that could be construed as a potential conflict of interest.

## Publisher’s note

All claims expressed in this article are solely those of the authors and do not necessarily represent those of their affiliated organizations, or those of the publisher, the editors and the reviewers. Any product that may be evaluated in this article, or claim that may be made by its manufacturer, is not guaranteed or endorsed by the publisher.

## Abbreviations

ASPLT, atypical spindle cell/pleomorphic lipomatous tumor; MRI, magnetic resonance imaging; IHC, immunohistochemistry; FISH, fluorescence *in situ* hybridization; MLS, myxoid liposarcoma; RB1, retinoblastoma; MDM2, mouse double minute 2 homolog; ALT/WDL, atypical lipomatous tumor/well-differentiated liposarcoma; SCL, spindle cell lipoma; DDLS, dedifferentiated liposarcoma; PLS, pleomorphic liposarcoma; PET-CT, Positron emission tomography-computed tomography; CDK4, cyclin-dependent kinase.
